# Evaluating the digital health technology landscape in sub-Saharan Africa and its implications for cardiovascular health

**DOI:** 10.1038/s44325-025-00055-9

**Published:** 2025-06-10

**Authors:** Omotayo A. Segun-Omosehin, Jesutofunmi A. Omiye, Aya Elalfy, Serin Moideen-Sheriff, Femi Kuti, Oluwatosin Omole, Kofoworola O. Ogunyankin, Ngozi Idemili-Aronu, John O. Olawepo, Echezona E. Ezeanolue, Demilade Adedinsewo

**Affiliations:** 1 St George’s University of London, London, UK; 2 University of Nicosia Medical School, University of Nicosia, Nicosia, Cyprus; 3 Department of Dermatology, Stanford University School of Medicine, Stanford, CA USA; 4 Department of Internal Medicine, Mayo Clinic, Jacksonville, FL USA; 5 Department of Cardiovascular Medicine, Mayo Clinic, Jacksonville, FL USA; 6 Reliance Health Inc, Austin, TX USA; 7 Maternal health Plus, Ekiti, Ekiti State Nigeria; 8 First Cardiology Consultants Healthcare, Ikoyi, Lagos Nigeria; 9 IVAN Research Institute, Enugu, Nigeria; 10 Department of Sociology/Anthropology and Community Medicine, University of Nigeria, Nsukka, Nigeria; 11 Department of Public Health and Health Sciences, Bouve College of Health Sciences, Northeastern University, Boston, MA USA; 12 Center for Translation and Implementation Research, College of Medicine, University of Nigeria, Nsukka, Nigeria; 13 Healthy Sunrise Foundation, Las Vegas, NV USA

**Keywords:** Cardiovascular diseases, Health care

## Abstract

Digital health technologies (DHTs) and novel artificial intelligence tools have already begun to transform healthcare in many parts of the world. Non-communicable diseases (NCDs) are on the rise in sub-Saharan Africa, with cardiovascular disease accounting for the largest NCD burden. Digital technologies can accelerate healthcare transformation and improve healthcare delivery in sub-Saharan Africa. This article explores digital technologies in sub-Saharan Africa and potential applications in the field of cardiovascular medicine.

## Introduction

In the last two decades, sub-Saharan Africa has experienced a remarkable digital transformation, largely driven by mobile technology penetration and innovation, with more than 80% of the population having a mobile phone subscription^1^. The rapid adoption of mobile technology resulted in sub-Saharan Africa effectively skipping the development and use of landline phones and transitioning into the digital age^2^. This phenomenon is often referred to as leapfrogging. The use of digital mobile and internet technologies has led to significant developments across financial, communication, and public sectors in multiple countries, with evidence of improved labor force participation and reduction in poverty rates, specifically in Nigeria and Tanzania^3^. Although the use of mobile communication technology has grown tremendously and approximately 84% of individuals in sub-Saharan Africa reside in locations with mobile internet services, internet use remains low, estimated at 22% in 2021^3^. As of 2023, mobile internet penetration was 25%^4^ and 37% for any internet use (including broadband)^5^, however, smartphone adoption is projected to reach 88% by 2030, and the use of mobile internet (4G and 5G) to reach 66%^4^.

Across the continent, many countries face significant healthcare challenges largely attributed to poor access to care, inadequate human resources, limited healthcare financing, ineffective use of available resources, and weak health information management systems^6–8^. Fortunately, health technology is ranked as one of the top five digital business sectors (based on total investment) in sub-Saharan Africa^3^, a region with an estimated population of 1.1 billion as of 2021 but expected to double by 2025. In addition, 2 of the 3 main categories of digital technology use identified as most promising in West Africa (internet of things and mobile telephones) have a huge potential to influence healthcare^9^. Studies in Africa have also demonstrated successful use of mobile health (mHealth) technologies in the healthcare domain, such as short message service (SMS) to improve oncology care coordination and education^10,11^, maternal health services^12–14^, post-surgical care^15^, and telemedicine for provision of mental health services^16^.

While communicable diseases have historically been the predominant driver of poor health in sub-Saharan Africa, there has been an increase in the occurrence of non-communicable diseases (NCD), with cardiovascular disease (CVD) accounting for the largest NCD burden. In fact, NCDs are projected to become the leading cause of death by 2030^17^. This shift is mostly due to prevalent cardiovascular risk factors, which include unhealthy diets, air pollution, and low levels of physical activity^17^. Up to 80% of global CVD-related deaths occurred in developing countries, with more than 1 million deaths reported in sub-Saharan Africa^18^. In response to the rising incidence and prevalence of CVD in sub-Saharan Africa and the huge unmet need for cardiovascular care^19^, it is imperative that novel and practical strategies are considered to mitigate disease burden and enhance cardiovascular care. Increased integration of digital tools in African healthcare systems can enable efficient access to patient information, expanded access to healthcare services through remote monitoring and telehealth, enhanced diagnostic capabilities, improved health literacy, and facilitate the adoption of healthy lifestyle changes. Moreover, the advent of healthcare artificial intelligence (AI) and machine learning (ML) algorithms presents substantial opportunities to transform health services in sub-Saharan Africa and promote health equity^20,21^. This progress has the potential to leapfrog healthcare in Africa into the 21st century.

Although many African nations have begun adopting digital health technologies (DHTs), challenges and opportunities for improvement remain. There have been a number of efforts by entrepreneurs, innovators, and other stakeholders to develop creative digital solutions to address local healthcare challenges, and the broad adoption of digital tools is predicted to significantly improve efficiency in healthcare spending without reducing quality by 2030. An analysis of the financial impact of DHTs showed an estimated savings of 400 million to 2.5 billion USD in Kenya, 700 million to 3.3 billion USD in Nigeria, and 1.9 billion to 11 billion USD in South Africa representing 4–14%, 4–10%, and 6–15% of projected healthcare spending in each country respectively^22^. These savings could potentially be redirected to other critical areas such as fair compensation for healthcare workers, improving health infrastructure, high-quality health services, and enhanced support for digital health tools, which is particularly important for financially constrained countries.

This article aims to explore and summarize the use of digital technologies for health care, emphasize relevant cardiovascular examples in various contexts within sub-Saharan Africa, highlight successful digital health initiatives, examine challenges and barriers to implementation, and provide potential solutions to effectively leverage digital technologies to transform cardiovascular care delivery.

## Digital technology services and applications for healthcare in sub-Saharan Africa

**Fig. 1:**
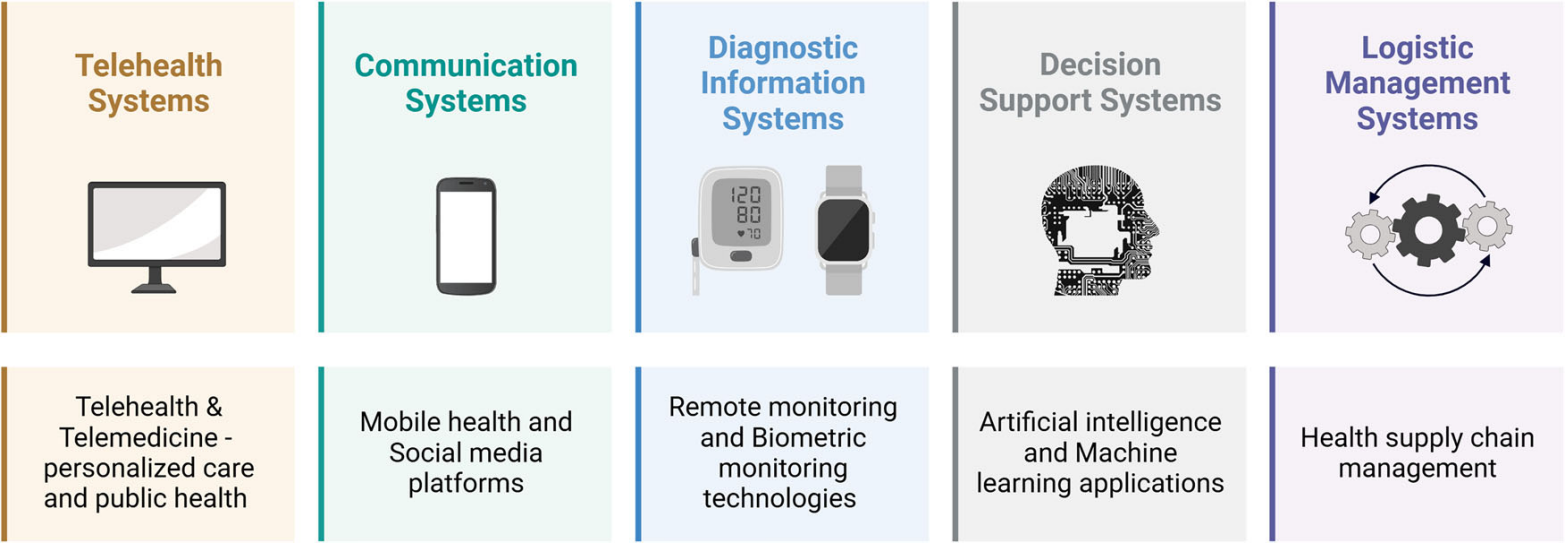
Digital Health Technology Applications and Services Impacting Cardiovascular Care in sub-Saharan Africa. This figure identifies 5 broad categories of digital health technologies (DHTs) in sub-Saharan Africa based on the World Health Organization (WHO) classification of DHTs. Underneath each panel (DHT group), a relevant clinical example is provided.

Digital technologies have the potential to transform health systems across the continuum of care for patients, from diagnostics to therapeutics and long-term care^15^. DHTs provide a potential solution to the large cardiovascular care access gap. This section broadly examines key DHT applications and services reported in sub-Saharan Africa and pragmatic use cases (Fig. 1) based on the World Health Organization (WHO) classification of DHTs^23^.

## Telehealth Systems

### Telehealth and telemedicine

Telehealth broadly refers to the use of electronic technologies to support and promote clinical care, health education, and public health, while telemedicine is considered a specific domain of telehealth centered around real-time interactive communication between patient and clinician located at a distant site^24^. The Coronavirus disease 2019 (COVID-19) pandemic catalyzed the adoption of telemedicine services across the world and in sub-Saharan Africa. Countries like Kenya and Rwanda launched dedicated telemedicine centers to manage COVID-19 cases, showcasing the ability to rapidly integrate and scale telemedicine services in resource-constrained environments^25^. Examples of digital health services to facilitate access to care and patient-doctor communications include ‘Babyl’ in Rwanda^26^, ‘Hello Doctor’ in South Africa^27^, ‘one2one’ and ‘Tremendoc’ in Nigeria^28,29^, and Reliance Health^30^, which provides telemedicine services, in-person primary care, and health plans in Nigeria. In Cameroon and Uganda, telemedicine services have been successfully utilized for transmission and remote interpretation of electrocardiograms (ECGs) and Echocardiograms^31^.

## Communication Systems

### Mobile health (mHealth)

Mobile health (mHealth) is one of the most popular forms of DHT in sub-Saharan Africa. Various studies have shown the utility of mHealth in increasing awareness of hypertension, promoting healthy behaviors, and providing clinical care^32,33^. A study in Nigeria showed that an mHealth application, which provided a platform for cardiologists to review patient blood pressure values and communicate with pharmacists, led to improved blood pressure control^32^. Similar results were replicated in Cameroon with study interventions and communication facilitated by phone calls, SMS, or voicemail^34^. The affordability and ubiquity of mobile devices in sub-Saharan Africa position mHealth as a unique tool for cardiovascular disease management^35^.

### Social media

Social media platforms provide instant access to health information for connected individuals across the globe, including sub-Saharan Africa, and can facilitate disease surveillance and public health education^36^. Healthcare professionals, institutions, and organizations often use platforms like Facebook, Twitter (X), LinkedIn, and Instagram to deliver cardiovascular health information, share preventive measures, advertise cardiovascular care services, and engage with the public on health-related issues^37,38^. The wide reach and real-time interaction offered by these platforms can be useful when designing population-based cardiovascular health interventions in resource-limited settings. However, there remains a significant concern regarding its misuse for disseminating false information and advice. This issue can be mitigated by encouraging the general public to rely solely on information from trusted sources, such as local health departments, reputable academic and healthcare institutions, as well as credible international sources that provide health-related information, including United Nations agencies such as the WHO, United Nations Children’s Fund (UNICEF), and United Nations Population Fund (UNFPA), among others.

## Diagnostic information systems

### Remote monitoring

The marked shortage of cardiologists to care for the millions of people in many regions of sub-Saharan Africa^39^ emphasizes the need for innovative solutions to aid diagnosis and treatment, including remote monitoring solutions. In Nigeria, the ratio of cardiologists relative to the total population is estimated at 1 to ~475,000 persons^40^. As of 2020, up to 18% of countries in sub-Saharan Africa did not have a registered cardiologist^39^. The Cardio Pad®, a portable ECG device which was developed and deployed in Cameroon utilizes non-specialist rural health workers to perform 3-lead, 6-lead, or 12-lead resting ECGs, and real-time continuous ECG monitoring using a tablet in remote villages. These ECGs can be transmitted wirelessly to cardiologists in urban centers via a mobile phone network for interpretation and management recommendations^41^. This technology facilitates early detection of acute coronary syndromes and arrhythmias and connects rural residents to specialized cardiovascular care. Other portable technologies evaluated for remote ECG monitoring include the AliveCor KardiaMobile, which has been approved for use in more than 25 countries^42^. One study evaluating the KardiaMobile device in Kenya showed a 98% acceptance rate, 100% study completion rate, and yielded a new diagnosis of atrial fibrillation in 8% of the study sample^43^.

### Biometric monitoring technologies (BioMETs)

This group of DHTs has become ubiquitous worldwide and is gaining popularity in many parts of sub-Saharan Africa as not just diagnostic tools, but also for health research, promoting healthy lifestyles, and managing chronic diseases^44–47^. Wearable BioMETs such as smart watches, bracelets, rings, patches, and garments paired with mobile phone apps enable users to monitor physical activity, sleep, track dietary intake, and other metrics that impact cardiovascular health^48^. Automated reminders from connected apps serve as motivation to continually engage in healthy behaviors with the goal of establishing good habits. Non-wearable BioMETs such as digital home blood pressure monitors, pulse oximeters, and glucose monitors provide users with the ability to monitor specific health measures at home to identify new conditions, improve treatment adherence, and guide medication management to prevent disease progression. Studies in Ghana and Nigeria have demonstrated some success in improving blood pressure control^49–51^. The use of virtual coaches, in addition to using BioMETs, to provide personalized advice and motivation to help individuals achieve health and fitness goals has also been shown to be successful in African countries^52^. These technologies empower users to take control of their health, make informed decisions, and adopt healthier behaviors, which are crucial for managing cardiovascular diseases^53^.

## Decision support systems

### Artificial intelligence and machine learning

The integration of AI in healthcare is poised to revolutionize patient care as we know it. The use of AI to guide echocardiographic image acquisition was shown to be feasible and effective in the identification of rheumatic mitral valve disease in Uganda^54^. AI-enabled ECG devices, including a digital stethoscope able to record a single lead ECG, were recently evaluated in a randomized clinical trial for peripartum cardiomyopathy (PPCM) screening in Nigeria^55^- known to have the highest reported PPCM incidence worldwide. The trial results showed that AI-guided screening doubled the detection of cardiomyopathy cases when compared to usual obstetric care^56^. AI-enabled portable ultrasound devices are also being evaluated in obstetric care to provide high-quality imaging and analysis, to aid in the early detection of complications and improve maternal and fetal outcomes^57–60^. The use of hand-held portable ultrasound devices, enabled with AI, has also been demonstrated to be useful for assessment, or left ventricular ejection fraction (LVEF)^61,62^ and detection of acute complications such as pericardial effusion^63^ and hemoperitoneum^64^. AI/ML applications have been developed to target various aspects of cardiovascular medicine ranging from disease detection to image acquisition and interpretation^65,66^. The list of approved AI-based cardiovascular health tools in the United States is rapidly expanding^67^, ranging from risk prediction and notification to diagnostic and disease monitoring software. However, only a limited number of these tools have demonstrated a direct impact on clinical outcomes within the context of randomized clinical trials^68^. Notable examples assessed in clinical trials include enhanced detection of cardiac dysfunction using AI-enabled ECGs in primary care settings^69^, as well as increased efficiency among cardiologists utilizing an AI-guided echocardiography interpretation workflow, which maintains interpretation quality^70^. Even fewer studies and implementation examples exist in sub-Saharan Africa, making it imperative to evaluate the clinical impact of AI tools for cardiovascular applications in African settings prior to widespread adoption. AI has immense potential to address barriers to care and extend specialized cardiovascular health services to underserved areas in sub-Saharan Africa by leveraging its ability to analyze large or complex datasets to identify patterns, predict cardiovascular risks, enable diagnosis in resource-limited settings, and support targeted screening and treatment interventions. AI can also enhance the efficiency of healthcare delivery services, promote equitable access to health across regions, and identify areas where further research is needed. The advent of generative AI, particularly large language models (LLMs), may yet provide novel cardiovascular care delivery solutions, but will require further exploration^71,72^.

## Logistics management information systems

### Health supply chain management

The use of digital platforms can improve access to essential medications and medical supplies, and many countries in sub-Saharan Africa have leveraged AI and DHT solutions to improve efficiency, reduce costs, and ensure the timely delivery of essential medical supplies. Key applications have been in the areas of demand forecasting to help optimize inventory levels and avoid shortages or overstocking. Other uses include inventory management, supply chain optimization, quality control, and risk management. Rwanda has implemented a nationwide digital network for medical supply distribution. This system allows the Ministry of Health to track the distribution of medical supplies in real time from the warehouse till it gets to the patient^73^. Additionally, the Transforming Rwanda Medical Supply Chain (TRMS) is an initiative funded by USAID that is expected to reduce procurement lead time by 75% and lower end-user markups from 66% to 40%^74^. LifeBank, an organization that originally aimed to reduce maternal deaths in Nigeria caused by blood shortages, developed an online platform to connect blood banks and hospitals^75,76^. Blood is requested online or via an app, and the blood product requests are then fulfilled expeditiously through motorcycle dispatch riders. Over time, LifeBank has evolved to also supply medical oxygen and has expanded into Kenya and Ethiopia^77^. Medical drones have also been used successfully in Malawi and Ghana to deliver emergency medicines and to improve service delivery^78^.

## Examples and pragmatic use cases of digital health technologies in sub-Saharan Africa

Across sub-Saharan Africa, numerous countries have implemented innovative digital health solutions aimed at improving healthcare access and patient outcomes. These initiatives span various aspects of healthcare delivery, from health data management and telemedicine to mobile diagnostics.

## Angola

The provision of self-paced and flexible electronic learning delivered through internet-enabled mobile devices for healthcare workers, called ‘Kassai’ has demonstrated the potential to use this innovative technology to improve capacity building across public and private sectors. ‘Kassai’ was developed by the Health for All project funded by USAID with the goal to provide education on Malaria management and other key health issues^79,80^. This technology can be expanded across various medical specialties, including cardiovascular medicine, for health system strengthening and continuing medical education.

## Ghana

‘mPedigree’, originally developed in Ghana, utilizes mobile phone SMS technology, to verify the authenticity of pharmaceutical products and provide instant feedback. It is now being used in other countries, including Kenya and Nigeria^81^. ‘Bisa’ is a telehealth platform developed to improve healthcare access in Ghana^82^. ‘Bisa’ offers mobile health care via phone or video calls, bi-directional communication with a health expert or physician, pharmaceutical services, an AI-powered chatbot for health-related questions, and personalized health and nutrition plans using AI algorithms^83^. The ‘AllRound Specialists Virtual Clinic’, established in 2021 and headquartered in Ghana, provides telemedicine services, including both text- and video-based consultations. The clinic also facilitates access to a range of medical specialists, encompassing cardiology and cardio-thoracic surgery^84^.

## Kenya

‘M-TIBA’, created in 2014, is a mobile phone health wallet that provides a platform for patients to save money for health care, pay for health services or health insurance premiums, receive aid to subsidize healthcare expenses, and for institutions to share and manage care utilization and claims data. The key goals of ‘M-TIBA’ is to improve healthcare delivery, provide a sustainable healthcare financing option in a transparent manner, and reduce individual out-of-pocket expenses^85^.

‘Afya Rekod’, founded in 2019, is a health technology company^86,87^ that provides a digital platform to support individual access to, storage of, and ownership of personal health records. It also offers an electronic health record system to health institutions and physicians, as well as telehealth solutions. It also hopes to utilize AI technology in the future to detect disease outbreaks and monitor disease progression^88^.

Several other digital health platforms utilizing AI technology exist in Kenya including: ‘Antara Health’^89^ which provides virtual primary care and connects patients with subspecialty care services; ‘SIHA AI’^90^, a mobile app that uses computer vision to improve microscopy and tissue pathology-based diagnostics; ‘Ilara health’^91^ which provides a health information management system and financing for diagnostic equipment and pharmaceutical supplies; ‘Afya Pap’^92^, a digital health companion which offers personalized health education and coaching for management of chronic disease; and ‘Zuri Health’ which utilizes a video-based AI solution (using photoplethysmography signals) for screening and monitoring of a range of biometric parameters and cardiovascular health metrics (including blood pressure, total cholesterol, and HBA1c)^93^. However, there is very sparse to no data regarding the effectiveness of these AI-solutions, nor are there published validation studies in Kenya or other African countries. Nonetheless, these technological solutions have the potential to support healthcare financing options, ensure comprehensive health record keeping, and facilitate diagnostic testing for cardiovascular care, as well as establishing follow-up care with primary care providers.

## Nigeria

Nigeria is the most populous country in Africa and leads the continent in both the number of digital technology start-ups and venture capital investments (reaching 1.8 billion USD in 2022)^94^. In Nigeria, health technology start-ups rank as the third most prevalent, after Fintech and e-commerce^94^. As such, there are several examples in Nigeria utilizing DHT’s in innovative ways that spans the whole spectrum of healthcare infrastructure and services from health education to delivery of clinical, laboratory, and pharmaceutical services, with some examples discussed below. ‘Cutica healthcare’ provides, via a web-based portal, curated information using short entertaining write-ups or skits, both in English and pidgin English, to address health topics or popular health-related myths and practices^95^. This platform can be utilized to enhance the dissemination of cardiovascular health education and prevention recommendations to the public, accommodating individuals of all educational backgrounds. ‘MyItura’ developed a mobile and web-based health information system that seamlessly connects independent healthcare providers (clinicians, laboratories, pharmacies) to patients. It offers solutions that providers can use to schedule appointments, document encounters, bill, order labs, and medications for patients. Patients can control access to their own records and manage their medical records as they move from one service to another, such that results and encounter details are available to any new caregiver. Providers benefit by getting access to an integrated health information system without needing to acquire any hardware or software, or storage infrastructure. ‘MyItura’ also provides an AI assistant (chatbot) directly to consumers for health-related questions^96^. ‘Reliance health’ provides a platform for patients to access health services, primary care, and pharmaceuticals through various health plans. They also offer employer-based health plans that allow access to their network of health services. Members are able to schedule physician consultations and receive prescriptions through a mobile health app^30^. ‘Wellahealth’ provides direct-to-consumer health insurance plans and digital health care services. It supports signing up for a plan using SMS based messaging. Some of the services include direct access to a clinician via a free messaging/video calling app – WhatsApp (owned by Meta and used by over 2 billion people worldwide) as long as the user has access to the internet or a mobile data plan; and an artificial intelligence chatbot that functions as medical assistant, available at any time to provide guidance and support to patients with chronic conditions^97^. ‘Mobihealth’ provides 24/7 telemedicine consultations through a mobile app and connects patients in Nigeria to clinicians (with various specialty expertise) within Nigeria or a different country, including the United States and United Kingdom, essentially allowing users to access healthcare experts globally^98^. ‘Vitira’ is a mHealth platform developed as part of a research study^99^ to securely store patient health records and share with care providers at the point of care without relying on electricity or internet^14^. ‘Vitira’ includes three components: an encrypted handheld smartcard, a mobile phone application, and an encrypted online database, but is not publicly available for clinical use yet. ‘AVIVA’ has been used to enhance the effectiveness of cervical cancer screening by connecting non-specialist health workers with gynecologic oncologists for teleconsultation and timely review of results^11^. The diverse range of telehealth platforms outlined above presents a significant opportunity to enhance access to cardiovascular care services, especially considering the limited availability of specialists within the country^40^.

‘Helpmum’ was founded to improve maternal and infant health in Africa by leveraging cost-effective digital solutions and artificial intelligence^100^. Digital services provided include AI-enabled child vaccination tracking and optimizing allocation^101^, health information chatbots, and educational programs for community birth attendants made available as pre-recorded videos on donated electronic devices and on YouTube^102^. The integration of AI-driven solutions has the potential to further enhance access to innovative cardiovascular care tools through portable technologies by facilitating their expedited adoption.

## Rwanda

‘Babyl Rwanda’, originally launched in 2016 to provide broad telehealth services for primary care, was successfully integrated into the existing health system, and grew rapidly to become the largest digital health service provider in Rwanda, providing up to 4000 medical consultations daily and reaching over a third of the adult population (2.5 million registered users) as of July 2023^26^. It provided a platform for patients to access qualified physicians and nurses, schedule appointments via mobile phones with automated dial codes, receive unique prescription codes via SMS, which could be redeemed at partner pharmacies, as well as access specialty care at partner health facilities using SMS-based referral codes^26^. However, ‘Babyl’ ceased operations in August 2023 due to financial difficulties experienced by its parent company^26^. Although ‘Babyl’ demonstrates feasibility and successful adoption of DHT for healthcare, it also underscores the potential challenge of ensuring long-term sustainability in resource-limited settings with insufficient healthcare financing. This initiative clearly illustrates that telehealth represents a viable solution for Rwanda’s healthcare resource challenges, given its widespread adoption over a short period of time. Moreover, its potential extends to cardiovascular care, contingent upon establishing a self-sustaining financing model to ensure long-term success.

## South Africa

With an average of 8 medical doctors per 10,000 persons^103^ as of 2021, the launch of the ‘Hello Doctor’ telemedicine platform eased physician workload and enhanced healthcare access in South Africa^81^, particularly in underserved areas. The ‘Hello Doctor’ system operates efficiently, with an average response time of 51 minutes, and a patient satisfaction survey revealed a 75% satisfaction rate^27^. ‘MomConnect’ is a flagship program launched by South Africa’s national department of health in 2014, designed to improve perinatal care, access, and outcomes among obstetric patients^104^. The program is offered through mobile phones and facilitates prenatal care registration, provision of maternal and infant health information, and an avenue to provide feedback regarding care quality^104^. The ‘MomConnect’ program was rapidly scaled with 50,000 women registered within the first month, and over a 3-year period, it attained the highest population-level coverage reported in the country (reaching a total of 1.7 million women, more than 60% of women attending prenatal care as of 2017)^105^. However, there are some concerns about the long-term self-sustainability of this initiative due to the high cost of maintenance estimated at approximately 1 million USD annually which is currently funded by the department of health, private companies, and philanthropic donors^106^. The two telemedicine examples mentioned above demonstrated rapid and widespread adoption, mirroring trends in other African countries, and suggest significant potential for delivering cardiovascular care, comparable to the success seen with primary and obstetric care services.

The initiatives discussed above emphasize the transformative power of digital technology in healthcare delivery across sub-Saharan Africa. By utilizing digital tools and online platforms, these countries are addressing critical healthcare challenges and enhancing access to care with the goal of improving patient outcomes.

### Challenges and barriers to DHT deployment and adoption

**Fig. 2:**
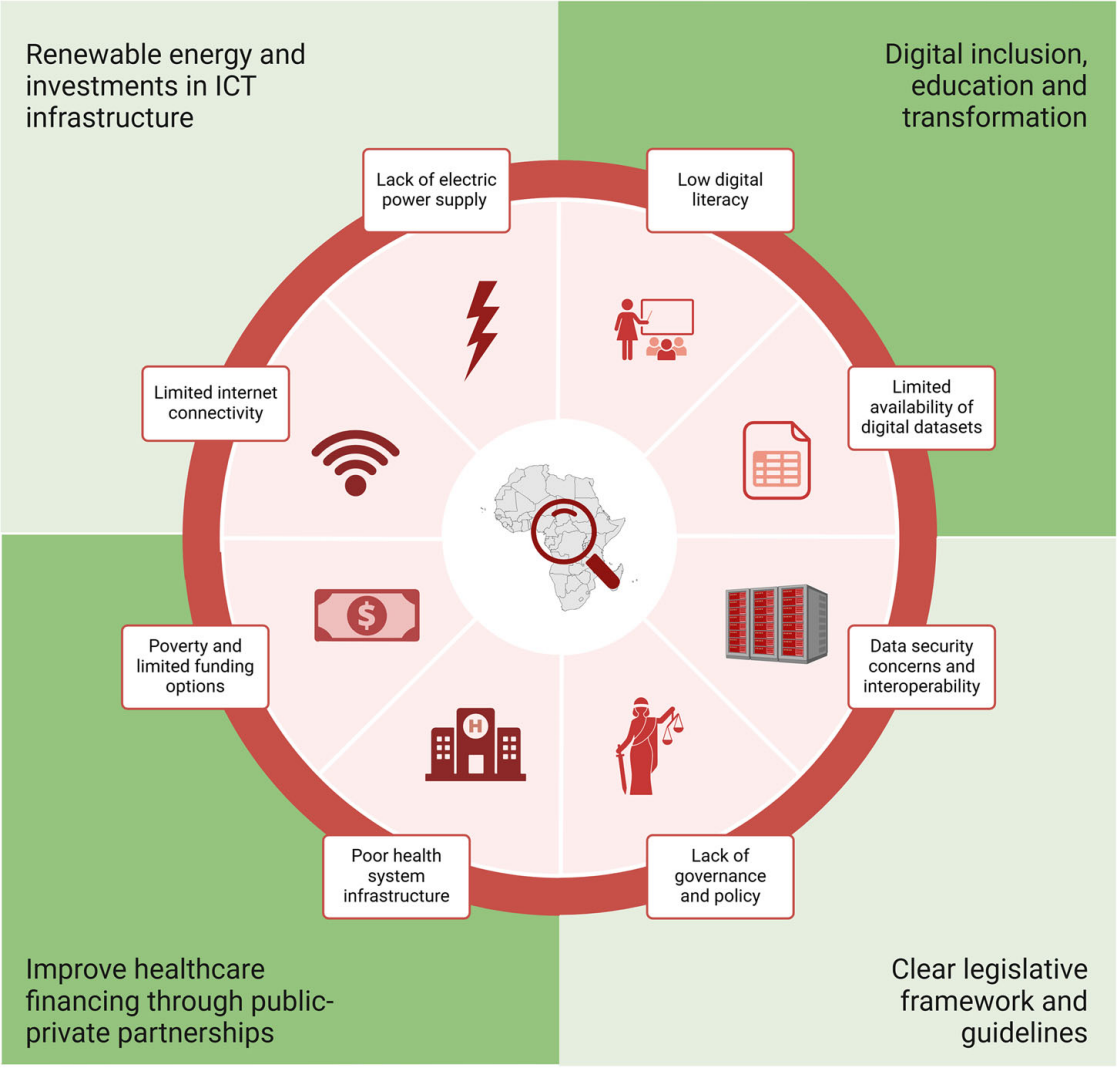
Barriers to Digital Health Technology Implementation and Proposed Solutions. The pie chart illustrates eight key barriers (in each slice) to digital health technology implementation in sub-Saharan Africa. The four quadrants surrounding the pie chart outline proposed solutions: (1) Renewable energy and investments in ICT infrastructure, (2) Digital inclusion, education, and transformation, (3) Improve healthcare financing, (4) Clear legislative framework and guidelines. ICT - Information and Communication Technology.

Despite the advances in sub-Saharan Africa’s digital health sector, numerous barriers and challenges must be addressed to realize its full potential (Fig. 2).

#### Unreliable power supply and limited connectivity

A major challenge is a lack of stable power supply, limited internet connectivity, and outdated infrastructure. Many digital technologies rely on access to stable electricity and or internet connectivity, however, the infrastructure for these varies and is more often lacking across the continent. An estimated 550 million people across the continent do not have reliable access to electricity and internet services^107^. Furthermore, USAID estimates that approximately 100,000 public health facilities in sub-Saharan Africa do not have reliable access to electricity and internet services^108^. The lack of dependable electric energy sources requires the use of alternate power sources such as diesel- or petrol-powered generators, which are costly to operate and maintain, and prone to frequent mechanical malfunction^109^. Recurrent electric power outage and intermittent use of backup generators leaves many medical devices susceptible to damage, and consequently, the need for frequent repairs or expensive replacements^109^.

#### Financial constraints and infrastructure gaps

Poverty, limited healthcare funding options, and poor health system infrastructure pose significant challenges to large-scale implementation of digital technologies in African countries. On average, many countries in sub-Saharan allocate between 5–6% of their gross domestic product (GDP) to healthcare^110^ compared to wealthier countries who spend from 10–16% of GDP on healthcare^111^. In South Africa, critical challenges to digital healthcare transformation identified were limited government funding, reliance on secondary infrastructure from private sector companies, geographical disparities in infrastructural availability, and insufficient resources to purchase hardware and software^112^. These issues are similar across other countries in sub-Saharan Africa. The recent suspension of USAID funding^113^, coupled with uncertainties regarding its future role in sub-Saharan Africa, has placed a spotlight on the substantial adverse health consequences associated with heavy reliance on foreign aid. This situation highlights the critical and urgent need for countries in sub-Saharan to develop a diverse portfolio of financial investments and to explore innovative alternate funding sources to support healthcare financing.

#### Low digital literacy among healthcare workers

In Ethiopia, it is estimated that about half of healthcare workers had sufficient digital literacy levels, based on a meta-analysis of 5 studies^114^. Factors associated with higher digital literacy were educational attainment, high income, perceived ease of use, and a positive attitude towards digital technologies. This digital literacy gap has also been observed among the general population in sub-Saharan Africa and many individuals do not have the necessary skills to use digital tools effectively^115,116^. A qualitative study of healthcare professionals working in primary care representing 13 countries in sub-Saharan Africa identified low digital literacy levels and access to technology as key challenges for patients to embrace DHTs^117^. Among healthcare workers, key challenges were limited financial and human resources, lack of support and training, and high staff turnover rates^117^. Socio-cultural beliefs also contribute to resistance to DHT adoption. One study evaluating barriers to the adoption of digital health tools found some rural communities in Botswana perceived technology to negatively interfere with the sanctity of human life^118^. In addition, a lack of knowledge and awareness of digital health tools contributes to patient reluctance in using DHTs^118^.

#### Limited availability of large digital datasets

The implementation of AI in healthcare holds numerous benefits for patients residing in sub-Saharan Africa, including improved diagnosis, prognosis prediction, and clinical decision support, which can improve healthcare efficiency^119^ and consequently reduce the workload burden on the limited number of healthcare professionals and specialists. However, the development of AI algorithms relies on the availability of large, complete, digital, and labeled clinical datasets, which are mostly non-existent in sub-Saharan Africa and can be very costly and time-consuming to curate prospectively. The limited use of electronic health records is a large contributor to the paucity of health data available to train AI models^66,120,121^. There is some concern that the use of AI algorithms developed outside Africa, trained with data from patient populations with likely different demographic and epidemiological characteristics, may lead to biased predictions and consequent risk of harm when deployed in Africa^120^. Furthermore, these algorithms may lack information on unique disease states and health conditions specific to certain regions in sub-Saharan Africa, leading to limited applicability and or relevance. However, a clinical trial evaluating an AI algorithm that was developed in a US-based patient population at the Mayo Clinic was shown to have strong performance among obstetric patients in Nigeria and improved the detection of left ventricular systolic dysfunction^56^. The study demonstrated the importance of validating AI algorithms in specific patient populations where it is intended for use. Thorough validation remains a key component of responsible artificial intelligence^122^ and can potentially address the challenge related to the paucity of large digital datasets in sub-Saharan Africa.

#### Governance and data security challenges

The complex and evolving regulatory and policy landscape in some countries has also limited the adoption of DHT and created uncertainty^123^. Lack of interoperability between different DHT systems and associated data has also been identified as a challenge^124^. Data security is also a major concern as it relates to electronic medical records and other protected health information collected with the aid of digital technologies. While the use of hardcopy paper records does not offer complete data security, recent data breaches at large public and private companies have rekindled public unease with the collection and storage of private digital health information^125^. Additionally, the legal framework supporting digital healthcare and data management is underdeveloped, with unclear guidelines for accountability related to AI use for healthcare^120^. Recently, the European Union AI Act was passed into law and addresses the use of AI for healthcare^126–129^. While data protection laws exist in some African countries – such as the Nigeria data protection regulation (NDPR)^130^ and Egypt’s Personal Data Protection Law (PDPL)^131^, clear regulations specific to the use of artificial intelligence in the context of healthcare data will be required to ensure adequate healthcare AI governance in sub-Saharan Africa.

Overall, despite the challenges and barriers identified that may hinder the implementation of DHTs in sub-Saharan Africa, its potential to positively transform healthcare delivery remains strong. Many countries are making concerted efforts to address these challenges, recognizing the massive potential benefits of digital health adoption to improve health outcomes.

### Potential solutions to address the digital health gap and future directions

Suggested recommendations to address existing challenges and enhance access to and availability of DHTs are identified below (Fig. 2).

#### Addressing power supply challenges

African countries need to consider alternative renewable energy options to address the lack of a stable electric power supply. Given the tropical climate in several regions of sub-Saharan Africa with many more hours of sunlight compared to other continents, there is substantial potential to harness solar energy as a major power source. Given the required infrastructure can be expensive to establish, government and private financial investments will be required. Companies such as Sun King (formerly Greenlight Planet), SunCulture and GridX Africa have already invested in solar energy solutions to support various sectors in multiple African countries^132–135^. Increased availability of affordable renewable energy sources can address the lack of a reliable electric power supply to support DHTs.

#### Strengthening healthcare financing through public-private partnerships and increased investment

Countries can improve healthcare financing by increasing national budgetary allocation to the healthcare sector and leveraging public-private partnerships and investment in healthcare. These investments can support the development of efficient and sustainable digital infrastructure and facilitate data curation efforts for AI model training and validation. Furthermore, public-private partnerships can enhance accessibility through cost-sharing measures as well as offering subsidized low-cost options to ensure that services remain affordable and accessible to patients across various income levels. A successful example is the Bill and Melinda Gates Foundation partnering with countries like Tanzania, Ethiopia, and Malawi to support health information gathering to guide public health programs^119^.

#### Digital inclusion, health education, and transformation

Investments in information and communications technology infrastructure will be critical to narrow the digital divide and connectivity access gap. Improving the availability of affordable, high-quality internet via government and private sector investments, along with thoughtfully crafted government policies and regulation, will be essential. Creation of public health campaigns to drive awareness of DHTs and improve public education on the use of these tools can enhance public digital literacy levels. Community-based educational/training programs can also leverage existing infrastructure and human resources such as community health workers, nurses, and digital health navigators^136^. Curation of electronic datasets through digitization of existing medical records and prospective development of electronic health data through national registries and surveys to facilitate research and development of context-appropriate AI/ML clinical decision support tools. DHT developers and companies also need to ensure these tools employ user-friendly interfaces to increase acceptability and uptake for individuals with limited digital literacy. Capacity building through targeted educational and training sessions for clinicians and health care professionals^137^ can support implementation and sustainability of DHTs.

#### Creating a regulatory landscape for responsible AI and digital health

The development of clear legislative frameworks and guidelines related to the use of DHTs and AI for healthcare is imperative. These should outline the roles and responsibilities of stakeholders, healthcare providers, and consumers. The Digital Health Act, enacted in Kenya in 2023, serves as an exemplary model by creating safeguards to ensure data privacy and security, facilitate data sharing for health-decision making, and prevent data misuse. It also established a digital health agency to ensure accountability^138^.

## Conclusion

The use of digital technologies and AI has the potential to improve cardiovascular care access and delivery in sub-Saharan Africa, ranging from enabling healthcare efficiency, facilitating health promotion, personalized medicine, population-based screening, and public health interventions. However, the infrastructure needed to realize the potential benefits of these tools will require investments by both public and private stakeholders. In addition, the need for prospective validation and implementation studies evaluating DHTs and healthcare AI tools in sub-Saharan Africa cannot be overemphasized. It is imperative that all stakeholders are engaged in this process, this includes governments and regulatory agencies, healthcare institutions, clinicians and allied health professionals, researchers, technology companies, and patients.

In summary, DHTs have a strong potential to enhance the quality and delivery of cardiovascular healthcare in sub-Saharan Africa. Although there are considerable challenges, we propose practical solutions to bridge the digital health gap and guide the development and implementation of digital health interventions, ensuring that the vast capabilities of these technologies are fully harnessed to advance cardiovascular care for all individuals.

## Data Availability

No datasets were generated or analyzed during the current study.

## Abbreviations

AI: Artificial intelligence
BioMETs: Biometric monitoring technologies
COVID-19: Coronavirus disease 2019
CVD: Cardiovascular disease
DHT: Digital health technology
ECG: Electrocardiogram
E-commerce: Electronic commerce
FINTECH: Financial Technology
GDP: Gross domestic product
HCV: Hepatitis C virus
LLM: Large language model
LVEF: Left ventricular ejection fraction
ML: Machine learning
mhealth: Mobile health
NCD: Non-communicable disease
SMS: Short message service
UNFPA: United Nations Population Fund
UNICEF: United Nations Children’s Fund
USAID: United States Agency for International Development
USD: Unites States Dollar
WHO: World Health Organization
4G: Fourth generation mobile phone technology
5G: Fifth generation mobile phone technology
